# The Effect of Interactions between Folic Acid Supplementation and One Carbon Metabolism Gene Variants on Small-for-Gestational-Age Births in the Screening for Pregnancy Endpoints (SCOPE) Cohort Study

**DOI:** 10.3390/nu12061677

**Published:** 2020-06-04

**Authors:** Rhodi E. Bulloch, Clare R. Wall, Lesley M. E. McCowan, Rennae S. Taylor, Claire T. Roberts, John M. D. Thompson

**Affiliations:** 1 Discipline of Nutrition and Dietetics, The University of Auckland, Auckland 1010, New Zealand; c.wall@auckland.ac.nz; 2 Department of Obstetrics and Gynaecology, The University of Auckland, Auckland 1010, New Zealand; l.mccowan@auckland.ac.nz (L.M.E.M.); r.taylor@auckland.ac.nz (R.S.T.); j.thompson@auckland.ac.nz (J.M.D.T.); 3 Robinson Research Institute, Adelaide Medical School, University of Adelaide, Adelaide 5005, Australia; claire.roberts@flinders.edu.au; 4 Flinders Health and Medical Research Institute, Flinders University, Bedford Park 5042, Australia; 5 Department of Paediatrics, Child and Youth Health, The University of Auckland, Auckland 1010, New Zealand

**Keywords:** folic acid, folate, small-for-gestational-age, genes, polymorphisms, pregnancy, supplementation, fetal growth

## Abstract

Small-for-gestational-age (SGA) is associated with significant perinatal morbidity and mortality. Our aim was to investigate gene-nutrient interactions between maternal one-carbon single nucleotide polymorphisms (SNPs) and folic acid supplement (FAS) use, and their association with SGA. Nulliparous New Zealand women with singleton pregnancy were recruited as part of the Screening for Pregnancy Endpoints prospective cohort study. Data on FAS use was collected via face-to-face interview at 15 weeks’ gestation; participants were followed prospectively and birth outcome data collected within 72 h of delivery. Participants were genotyped for MTHFR 677, MTHFR 1298, MTHFD1 1958, MTR 2756, MTRR 66 and TCN2 776 SNPs. Genotype data for at least one SNP was available for 1873 (93%) of eligible participants. Analysis showed a significant SNP-FAS interaction for MTHFR 1298 (*p* = 0.020), MTHFR 677 (*p* = 0.019) and TCN2 776 (*p* = 0.017) in relation to SGA: MTHFR 1298 CC variant non-FAS users had an increased likelihood [Odds Ratio (OR) = 2.91 (95% Confidence Interval (CI) = 1.52, 5.60] compared with wild-type (MTHFR 1298 AA) FAS users. MTHFR 677 variant allele carrier (MTHFR 677 CT + MTHFR 677 TT) non-FAS users had an increased likelihood [OR = 1.87 (95% CI = 1.21, 2.88)] compared to wild-type (MTHFR 677 CC) FAS users. TCN2 776 variant (TCN2 776 GG) non-FAS users had an increased likelihood [OR = 2.16 (95% CI = 1.26, 3.71)] compared with wild type homozygote + heterozygote (TCN2 776 CC + TCN2 776 CG) FAS users. No significant interactions were observed for MTHFD1 1958, MTR 2756 or MTRR 66 (*p* > 0.05). We observed an overall pattern of FAS attenuating differences in the likelihood of SGA seen between genotype groups in FAS non-users. Future research should focus on how intake of other one-carbon nutrients might mediate these gene-nutrient interactions.

## 1. Introduction

Small for gestational age (SGA), defined as a birthweight below the 10th centile, is associated with significant perinatal morbidity and mortality, and there are currently few preventive strategies [1,2]. Fetal growth and SGA are influenced by a number of factors including maternal socio-demographic characteristics, nutritional intake and status, and genetics [3,4,5].

Folate, a water soluble B-vitamin, is essential for fetal and placental growth and development, through its role in one-carbon metabolism, and DNA replication, synthesis and methylation [5,6,7,8,9,10]. Folate requirements are increased during pregnancy [8,10,11,12], and adequate maternal folate intake pre-conception and during pregnancy is important for healthy development of the placenta and fetus [8,10,13,14]. Previous systematic reviews indicate a positive association between maternal prenatal folic acid supplementation (FAS) use and fetal growth [15,16]. The World Health Organization Antenatal Care Guidelines include the following recommendation: “Daily iron and folic acid supplementation with 30 mg to 60 mg of elemental iron and 400 µg (0.4 mg) of folic acid is recommended for pregnant women to prevent maternal anaemia, puerperal sepsis, low birth weight, and preterm birth”. [14] (p. 23) and that “Folic acid should be commenced as early as possible (ideally before conception) to prevent neural tube defects” [14] (p. 23).

Functional polymorphisms of genes encoding enzymes involved in folate-mediated one-carbon metabolism can cause disturbances in folate status due to a reduction in enzyme activity [6,8,17,18]. Women with these genetic polymorphisms are at risk of low folate status and adverse pregnancy outcomes including poor fetal growth and SGA, although findings are inconsistent [6,17,19,20,21,22,23,24,25].

Genetic polymorphisms interact with environmental factors to modify disease risk [18,26]. Maternal folate intake plays an important role in the phenotypic expression of mutations in the folate metabolic pathway [18,27]. Differences in folate intakes (including FAS, dietary folate and folic acid from fortified foods) between populations may account for the differences in effects of polymorphisms on outcomes [18,22], which are especially prevalent when folate intake is low [18]. Women with polymorphisms in the folate-mediated one-carbon metabolic pathway may have an increased folate requirement [19], and may benefit from an increased dose and duration of FAS in pregnancy [28].

Few studies have examined the influence of folate gene-nutrient interactions on fetal growth outcomes, with inconsistent findings [23,29]. The aim of this study was to use data from the New Zealand participants in the Screening for Pregnancy Endpoints (SCOPE) prospective cohort study to investigate how gene-nutrient interactions between maternal polymorphisms of the folate mediated one-carbon metabolic pathway and maternal FAS are associated with SGA.

Specific objectives were to: (1) describe the distribution of the maternal SNP genotypes in our cohort, specifically: MTHFR 677 (rs1801133), MTHFR 1298 (rs1801131), MTHFD1 1958 (rs2236225), MTR 2756 (rs1805087), MTRR 66 (rs1801394), TCN 776 (rs1801198); (2) examine the association between these maternal SNPs and SGA; and (3) determine whether there is a genotype-FAS use interaction in relation to SGA.

## 2. Materials and Methods

### 2.1. Participant Recruitment and Selection

This study is a secondary analysis using data from New Zealand participants of the SCOPE international multi-centre prospective cohort study. SCOPE was conducted in nulliparous women with singleton pregnancies, with the primary aim of producing clinically useful screening tests to detect nulliparous pregnant women at high risk of adverse birth outcomes of preeclampsia, spontaneous preterm birth and/or SGA (www.scopestudy.net). The SCOPE study was registered with the Australia New Zealand Clinical Trials Registry (ID 82254) [30]. Participants from Auckland (New Zealand) were recruited between 2004 and 2009. Details of SCOPE study methodology have been published in detail previously [31]. Women were excluded if they were considered at high risk of preeclampsia, spontaneous preterm birth or SGA birth due to major medical conditions or medical/gynaecological history [31].

### 2.2. Ethics

Ethical approval was obtained [New Zealand AKX/02/00/364–23 April 2003] and all participants provided written informed consent for inclusion before they participated in the study. The study was conducted in accordance with the Declaration of Helsinki.

### 2.3. Sociodemographic, Lifestyle and FAS Data Collection

Participants who agreed to take part were interviewed face-to-face and examined by a trained research midwife at a research visit conducted at 15 (±1) weeks’ gestation. A detailed sociodemographic, family and medical history, as well as lifestyle data, were collected via an interview-administered questionnaire at this research visit. Questions on maternal FAS use were included in the questionnaire [31,32]. Information on FAS use covered the pre-conception time-period (defined as one month before conception), the first trimester, and current supplement use (at 15 (±1) weeks’ gestation). Participants were asked to bring their pregnancy supplements to the SCOPE visit, to verify their folic acid content. Maternal FAS use (Yes/No) at 15 (±1) weeks was chosen as the FAS exposure variable in this study, as maternal report of FAS use at 15 (±1) weeks has previously been confirmed by plasma folate levels in the SCOPE Auckland Cohort [32], suggesting that maternal report of FAS with researcher verification was a reliable proxy of FAS intake [32]. FAS users for this analysis were therefore defined as those participants who took FAS at 15 weeks’ gestation vs. those who did not take FAS at 15 weeks’ gestation. Data were entered at the time of the interview by trained research midwives into an internet-accessed, password-protected centralised database with a complete audit trail (MedSciNet AB, Stockholm, Sweden).

### 2.4. Single-Nucleotide Polymorphisms

Six SNPs were included in this study: MTHFR 677 (rs1801133), MTHFR 1298 (rs1801131), MTHFD1 1958 (rs2236225), MTR 2756 (rs1805087), MTRR 66 (rs1801394), TCN2 776 (rs1801198). Supplementary Table S1 describes the chromosomal location of each SNP, the enzyme encoded by the associated gene, enzyme function and effect of the polymorphism [6,22,33,34,35,36,37,38,39].

### 2.5. Analysis of Single Nucleotide Polymorphisms

Blood was drawn from participants at the 15 (±1) weeks’ SCOPE research visit by trained research midwives, into purple-top EDTA-vacutainers (6 mL per vacutainer) and centrifuged at 3000 rpm (2400× *g*) for 10 min at 4 degrees Celsius. Vacutainers and second spin tubes were kept in ice until aliquots and buffy coat were completed. EDTA plasma was pipetted into lavender-capped barcoded cryotubes in 250 uL quantities without disturbing the buffy coat, leaving a small amount of plasma on top. A new sterile ‘transfer’ pipette tip was then used to carefully remove the white cells (buffy coat) just above the red blood cells (interface between the plasma and red blood cells). Buffy coat was transferred into a sterile brown-top barcoded cryotube which was centrifuged at 3000 rpm (2400× *g*) for 10 min at 4 degrees Celsius. With a new sterile pipette tip any residual supernatant was aspirated from the buffy coat and discarded. A brown cap was fitted to the cryotube, barcode scanned immediately into SCOPE database and cryotube of buffy coat was placed in −80 degrees Celsius freezer for storage. Buffy coat samples stored in a unique barcoded aliquot were drawn from storage, placed into dry ice and couriered to the SCOPE Adelaide research group.

DNA was extracted from maternal buffy coat samples using a QIAamp 96 DNA blood kit (QIAGEN) using the manufacturer’s instructions, by the Australian Genome Research Facility (AGRF, Adelaide) and was then transported to AGRF Brisbane for multiplex genotyping using the Sequenom Mass Array System. For quality control all samples were also genotyped for Amelogenin to ensure the sex of the sample was correct.

### 2.6. Outcomes and Definitions

Participants were followed prospectively from the 15 (±1) weeks’ gestation research visit until delivery. Pregnancy outcome data and birth size measurements were collected, usually within 72 h of birth. The primary outcome of this study was SGA, defined as a birth weight <10th customised birthweight centile (adjusted for maternal ethnicity, maternal booking weight and height, and infant sex and delivery gestation) [40].

### 2.7. Statistical Analysis

Analyses were carried out using STATA Version 5 (StataCorp). Maternal characteristics are reported as counts (*n*) and percentages (%) for categorical variables, and means and standard deviations (SD) for continuous variables. Chi-square tests were used to examine whether genotype distributions were in Hardy-Weinberg equilibrium, using the **genhw** command in STATA Version 5 (StataCorp). Logistic regression analysis was used to investigate the association between maternal SNPs (genotype status) and SGA, using the genotype model of inheritance. This model compares heterozygous and homozygous variant genotypes separately to the wild-type homozygote genotype (reference category). To investigate the SNP-FAS interaction on SGA, logistic regression analysis was used, which included a genotype-FAS interaction term. FAS use was chosen as the FAS reference category. Homozygous wild-type genotype was chosen as the SNP reference category. Interaction analysis was conducted under the genotype model of inheritance. SNP genotypes were then combined into dominant (homozygous wild-type (reference category) vs. heterozygous + homozygous variant genotypes) or recessive (homozygous wild-type + heterozygous variant genotypes (reference category) vs. homozygous variant genotype) models in order to investigate associations further [41,42]. Combining genotype groups into recessive or dominant models increases the ability to detect SNP-disease associations when the assumed inheritance model is the true one [42]. To check for any potential confounding effects of ethnicity on gene-nutrient interactions, a sensitivity analysis was conducted using the largest maternal ethnic group in the sample (NZ/Other European subjects). Statistical significance was defined at the 5% level; odds ratios (for categorical outcomes) were estimated using logistic regression and reported with 95% confidence intervals.

## 3. Results

### 3.1. Description of Sample

**Figure 1:**
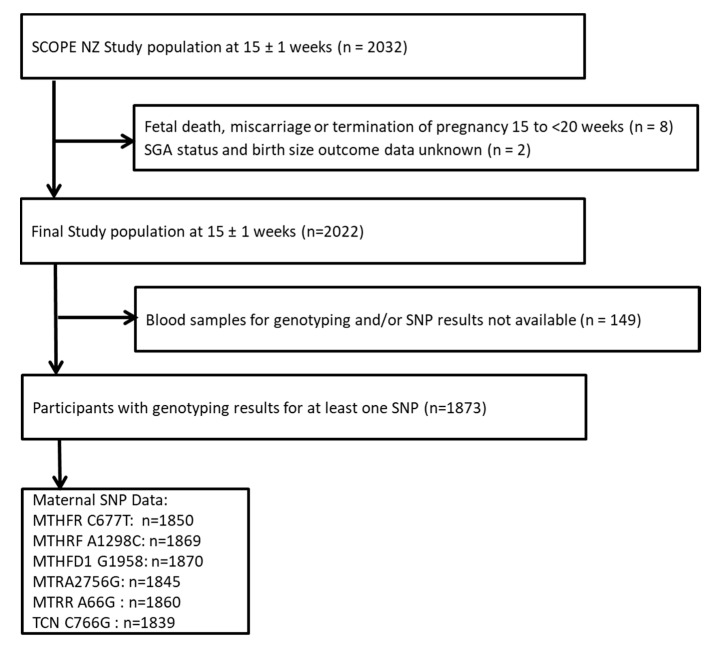
Participant selection flow chart.

The flow of participant selection is shown in Figure 1. There were 2022 participants eligible for inclusion in this analysis, and blood samples for maternal genotyping and results of at least one SNP were available for *n* = 1873 (92.6%) of eligible participants. Figure 1 gives the number of participants who were genotyped for each of the six SNPs.

Table 1 describes the characteristics of participants in this sample (*n* = 1873), including FAS use at 15 (±1) weeks’ gestation, and pregnancy outcomes. Full details of FAS use, dose and plasma folate in the SCOPE New Zealand cohort have previously been described [32]. Seventy-three percent of this sample reported taking a FAS supplement at the first research visit (15 (±1) weeks’ gestation). The prevalence of SGA in this sample was 10.1% (*n* = 189). Mean birth weight was 3414 g (s.d. = 570 g).

### 3.2. Genotype Distribution and Allele Frequency

The distributions of maternal genotypes for all six SNPs were in Hardy-Weinberg equilibrium (*p* > 0.05), and are shown in Table 2. The allele frequencies for the variant allele were as follows: 30.8% for MTHFR 677, 29.3% for MTHFR 1298, 42.2% for MTHFD1 1958, 19.2 for MTR 2756, 51.8 for MTRR 66 and 45.6 for TCN2 766. Supplementary Table S2 gives the genotype and allele frequencies according to each ethnic group included in this sample.

### 3.3. Associations between Maternal Polymorphisms and SGA

No statistically significant associations (*p* < 0.05) were observed between maternal genotype status and SGA for any of the six SNPs studied (Table 2).

### 3.4. SNP-FAS Interactions and SGA

The associations between maternal SNP-FAS interaction and SGA are shown in Table 3. Under the genotype model of inheritance, analysis showed a significant interaction between FAS and MTHFR 1298 (*p* = 0.020), with MTHFR 1298 CC genotype (homozygous variant) non-FAS users having an increased likelihood of SGA [Odds Ratio (OR) = 2.91 (95% Confidence Interval (CI) = 1.52, 5.60] compared to wild-type (AA) FAS users. Under the dominant model of inheritance, analysis showed a significant interaction between FAS and MTHFR 677 (*p* = 0.019), with carriers of the variant allele (CT + TT) who did not use FAS having an increased likelihood [OR = 1.87 (95% CI = 1.21, 2.88)] of SGA compared to wild-type (CC) FAS users. Under the recessive model of inheritance, a significant interaction was observed for MTHFR 1298 (*p* = 0.005), with results showing a significantly higher likelihood of SGA in the homozygous variant group (CC) who did not use FAS, in comparison with the wild type homozygotes (AA) + heterozygotes (AC) who used FAS [OR = 3.11 (95% CI = 1.66, 5.85)]. Under the recessive model of inheritance, a significant interaction was also observed between FAS and TCN 2 776 (*p* = 0.017), with TCN2 776 variant homozygote (GG) non-FAS users having an increased likelihood of SGA [OR = 2.16 (95% CI = (1.26, 3.71] compared with wild type homozygote + heterozygote (CC + CG) FAS users. No statistically significant interactions were observed for MTHFD1 1958, MTR 2756 or MTRR 66 (*p* > 0.05) (Table 3).

A sensitivity analysis conducted using NZ/Other European participants (the largest ethnic group in the sample) showed that interactions did not differ notably from those of the total sample, and the statistical significance and effect size of the three interactions described above remained consistent.

**Figure 2:**
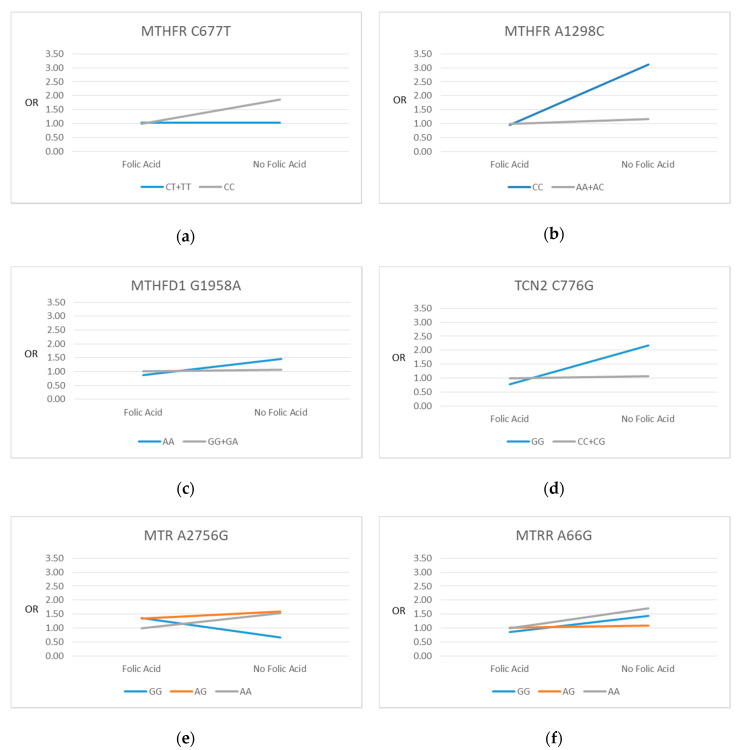
Plots of regression odds ratios (OR): SNP vs. SGA by maternal folic acid supplement use at 15 weeks’ gestation. (**a**) MTHFR C677T; (**b**) MTHFR C1298A; (**c**) MTHFD1 G1958A; (**d**) TCN2 C776G; (**e**) MTR A2756G; (**f**) MTRR A66G. Folic Acid: reference category (defined as those who took folic acid supplements). Reference genotype category is shown in grey.

Figure 2 gives a visual representation of the directions of the SNP-FAS interactions on SGA for each of the six SNPs. Although not all interactions were significant, FAS use showed a general pattern of attenuating the differences in the likelihood of SGA seen between genotype groups who did not use FAS.

## 4. Discussion

In this study of nulliparous pregnant women living in New Zealand, significant gene-nutrient interactions in relation to SGA were observed between three maternal one-carbon metabolism gene polymorphisms—MTHFR 677, MTHFR 1298, and TCN2 776 and maternal FAS use at 15 (±1) weeks’ gestation. Although interactions for the other SNPs were not significant, we observed a general pattern of FAS attenuating the differences in the likelihood of SGA seen between genotypes in FAS non-users. The exception was for the MTR 2756 homozygous variant (GG) genotype, which showed a non-statistically significant reduction in SGA in FAS non-users in comparison with FAS users. However, as the prevalence of the MTR 2756 GG genotype was very low in this sample (3.8%), this may be a chance finding, and this genotype and its interaction with folate requires investigation in future studies.

Few previous studies have examined the effect of folate gene-nutrient interactions on birth size-parameters or SGA. To the best of our knowledge, this is the first study to examine maternal gene-nutrient interactions between FAS use (supplement form) and these six SNPs of the folate-mediated one-carbon metabolic pathway, and their effects on SGA.

One previous study that examined gene-nutrient interactions between maternal total folate intake (dietary folate, folic acid from fortified foods and FAS) and MTHFR 677, MTHFR 1298, MTR 2756 and MTRR 66 polymorphisms and SGA [23] found a statistically significant lower odds of SGA in MTHFR 1298 AA wild-type participants vs. variant homozygote (CC) Caucasian participants in the lowest quartile of total folate intake (OR = 0.3; 95% CI: 0.1–0.9). Although the study focused on total folate intake, the finding of a significant result for MTHFR 1298 aligns with our finding of a significantly higher likelihood of SGA seen in MTHFR 1298 variant CC participants who did not use FAS, in comparison with wild-type AA FAS users. Although Engel and colleagues did not observe statistically significant associations for MTHFR 677, MTR 2756 and MTRR 66 in the lowest quartile of folate, point estimates of the effect sizes were in the same direction as seen in MTHFR 1298 (OR: 0.6, 0.3 and 0.6 respectively) [23]. A second folate gene-nutrient interaction study found no significant interaction between maternal dietary folate and MTHFR 677 or 1298 polymorphisms on anthropometric birth parameters (*n* = 231) [29]. This is in contrast to our study (*n* = 1873) which showed a significant interaction effect for both of these polymorphisms. The difference between findings may be due to Torres-Sánchez and colleagues using dietary folate in their gene-nutrient analysis, whereas the present study focused specifically on FAS use.

A number of biological mechanisms are likely to explain our findings of an interaction effect between FAS and maternal genotype on SGA. During pregnancy, folate is important for fetal growth and development through its role in one-carbon metabolism, DNA synthesis, replication and methylation, and requirements are increased, due to rapid placental and fetal growth [43]. Folate plays an essential role in embryonic and fetal development [44]. Adequate maternal folate status is important during pregnancy to ensure optimal maternal folate status and homocysteine levels [15]. Maternal plasma folate and homocysteine levels are influenced by both folate intake and polymorphisms of the folate-mediated homocysteine metabolic pathway [45]. These polymorphisms can cause disturbances in folate metabolism, one-carbon metabolism, DNA synthesis and methylation [6,17,35,46], which can in turn impair fetal growth and development. An increased supply of folic acid might be required for one-carbon metabolism in the presence of these polymorphisms [44]. FAS is likely to attenuate the effect of these SNPs on pregnancy outcomes by supplying additional folic acid and therefore an increased availability of one-carbon groups for the conversion of homocysteine to methionine, helping to maintain normal homocysteine levels [15,20,47]. Previous research shows that folic acid (in supplement form) interacts significantly with the MTHFR 677 polymorphism to influence homocysteine levels [19]. The authors concluded that MTHFR 677 homozygous variant (TT) genotype carriers need increased intakes of total folate compared to those with CC and CT genotypes, to maintain similar plasma homocysteine levels [19]. Similarly, a review by Hiroaka and colleagues (2017) of studies that examined differences in maternal FAS intake and serum folate and homocysteine levels, found that differences in mean levels at baseline seen between all three genotype groups of the MTHFR 677 polymorphism were attenuated in the presence of FAS [45,48,49,50].

A recent review by Zinck and MacFarlane suggests that limitations of previous gene-nutrient interaction studies include small sample sizes, and lack of dietary supplement data [18]. SCOPE contains time-specific data on maternal FAS use in a relatively large sample size. Participants were followed prospectively from 15 (±1) weeks’ gestation until after delivery, allowing capture of FAS data before birth outcomes. When collecting FAS data, participants brought their supplements to the research visit so that the investigator could verify details and record brand names and details directly into the study database. Reported FAS use was previously confirmed by plasma folate analysis in the NZ SCOPE cohort [32].

The current study has a number of limitations that must be considered in the context of our findings. SCOPE includes an opportunistic population of nulliparous participants from Auckland, New Zealand, and was predominantly of NZ/Other European ethnicity, and therefore not representative of New Zealand’s overall ethnic composition. This did not allow separate investigation of SNP-SGA effects in each ethnic group, due to small numbers of other ethnic groups included in the sample. However, we hypothesised that the biological effects of the FAS-SNP interaction would likely be similar across ethnic groups, and sensitivity analysis showed results remained consistent when limited to European participants. Although numbers of non-European ethnic groups in our study were small, our results contribute data on the distribution of the six included SNPs in the New Zealand population. Further research is required to describe these genotype distributions in larger samples of NZ ethnic groups. An important limitation of our study is that SCOPE study data collection did not include a comprehensive food frequency questionnaire or 24-h dietary recall, and we were therefore unable to estimate participant total dietary folate intake. Future interaction studies should examine the influence of FAS genotype interactions across groups with different background dietary folate intakes, to investigate further the FAS-polymorphism interaction association with fetal growth parameters. Folic acid in supplements is a synthetic form and is different to dietary folate. Folic acid in supplement form is more bioavailable than folate occurring naturally in food, hence the two might have different effects in the presence of folate enzyme SNPs.

## 5. Conclusions

Significant gene-nutrient interactions in relation to SGA were observed for FAS and three gene polymorphisms of the one-carbon metabolic pathway. FAS showed an overall pattern of attenuating differences in the likelihood of SGA seen between genotype groups in FAS non-users. This highlights the importance of ensuring adequate folic acid intake for women with these polymorphisms. Further research is required to investigate how differences in total folate intake (from dietary sources, food fortification and supplements) influence this interaction. Future research should focus on how folate biomarkers, FAS at other time points (such as pre-conception) and intake of other nutrients involved in one carbon metabolism (B12, choline, biotin) might mediate these interactions.

## Figures and Tables

**Table 1:** Participant Characteristics.

Characteristic	Mean ± SD or *n* (%)
Total *n* (%)	1873 (100)
Maternal age (years)	30.4 ± 4.7
Ethnicity:	
NZ/Other European	1577 (84.2)
Māori	61 (3.3)
Pacific	35 (1.9)
Asian	97 (5.2)
Indian	74 (4.0)
Other Non-European	29 (1.6)
Socioeconomic Index *	48 ± 15
Education > 12 years	1160 (64.8)
Marital partner (yes)	1811 (96.7)
BMI at 15 weeks’ research visit (kg/m^2^)	24.8 (±4.2)
Smoking (at 15 weeks’)	73 (3.9)
Folic Acid Supplement Users ^$^	1367 (73.0)
Folic Acid Supplement Dose (µg/day)	564 (14) ^¥^
Plasma Folate (nmol/L) ^§^	48.6 (1.6) ^¥^
Fetal Sex:	
Male	973 (52.0)
Female	900 (48.0)
Final Delivery Gestation (weeks)	39.6 (±2.1)
Birthweight (grams)	3414 (±570)
Customised Birthweight Centile	49 (±29)
SGA	189 (10.1)
Spontaneous Preterm Birth	77 (4.1)

SD, standard deviation; BMI, body mass index; SGA, small-for-gestational-age. * Socioeconomic Index scale ranges from a score of 10 to 90, with a higher score representing a higher socio-economic status) (35). ^$^ Defined as participants taking folic acid supplements at 15 weeks’ gestation. ^§^ Non-fasting plasma folate at 15 (±1) weeks’ gestation (assessed by microbiological assay [32]); geometric mean. ^¥^ Mean (SE).

**Table 2:** Maternal Genotype Frequency and Association between SNPs and SGA Risk in New Zealand SCOPE Participants.

SNP:	Total *n* (%)	SGA *n* (%)	OR (95% CI)	*p*-Value (*p* > z)	*p*-Value for Overall Genotype Effect
**MTHFR C677T**	1850 (100)				
**Genotype:**					0.457
MTHFR 677 CC (Ref)	887 (48.0)	96 (10.8)	1.00 (Ref)		
MTHFR 677 CT	786 (42.5)	15 (9.3)	0.84 (0.61–1.16)	0.299	
MTHFR 677 TT	177 (9.6)	73 (8.5)	0.76 (0.43–1.35)	0.352	
**Dominant Model**					0.227
MTHFR 677 CC (Ref)	887 (48.0)	96 (10.8)	1.00 (Ref)		
MTHFR 677 CT + TT	963 (52.1)	88 (9.1)	0.83 (0.61–1.12)	0.227	
**MTHFR A1298C**	1869 (100)				
**Genotype:**					0.141
MTHFR 1298 AA (Ref)	932 (49.9)	93 (10.0)	1.00 (Ref)		
MTHFR 1298 AC	778 (41.6)	72 (9.3)	0.92 (0.67–1.27)	0.614	
MTHFR 1298 CC	159 (8.5)	23 (14.5)	1.53 (0.93–2.49)	0.092	
**Recessive Model**					0.055
MTHFR 1298 AA + AC (Ref)	1710 (91.5)	165 (9.6)	1.00 (Ref)		
MTHFR 1298 CC	159 (8.5)	23 (14.5)	1.58 (0.99–2.53)	0.055	
**MTHFD1 G1958A**	1870 (100)				
**Genotype:**					0.766
MTHFD1 1958 GG (Ref)	621 (33.2)	67 (10.8)	1.00 (Ref)		
MTHFD1 1958 GA	920 (49.2)	91 (9.9)	0.91 (0.65–1.27)	0.569	
MTHFD1 1958 AA	329 (17.6)	31 (9.4)	0.86 (0.55–1.35)	0.510	
**Recessive Model**					0.650
MTHFD1 1958 GG + GA (Ref)	1541 (82.4)	158 (10.3)	1.00 (Ref)		
MTHFD1 1958 AA	329 (17.6)	31 (9.4)	0.91 (0.61–1.37)	0.650	
**MTR A2756G**	1845 (100)				
**Genotype:**					0.438
MTR 2756 AA (Ref)	1206 (65.4)	116 (9.6)	1.00 (Ref)		
MTR 2756 AG	569 (30.8)	66 (11.6)	1.23 (0.90–1.70)	0.200	
MTR 2756 GG	70 (3.8)	7 (10.0)	1.04 (0.47–2.33)	0.916	
**Recessive Model**					0.945
MTR 2756 AA + AG (Ref)	1775 (96.2)	182 (10.3)	1.00 (Ref)		
MTR 2756 GG	70 (3.8)	7 (10.0)	0.97 (0.44–2.16)	0.945	
**MTRR A66G**	1860 (100)				
**Genotype:**					0.714
MTRR 66 AA (Ref)	450 (24.2)	50 (11.1)	1.00 (Ref)		
MTRR 66 AG	892 (48.0)	88 (9.9)	0.88 (0.61–1.26)	0.478	
MTRR 66 GG	518 (27.9)	50 (9.7)	0.85 (0.57–1.29)	0.457	
**Dominant Model**					0.418
MTRR 66 AA (Ref)	450 (24.2)	50 (11.1)	1.00 (Ref)		
MTRR 66 AG + GG	1410 (75.8)	138 (9.8)	0.87 (0.62–1.22)	0.418	
**TCN2 C766G**	1839 (100)				
**Genotype:**					0.420
TCN2 766 CC (Ref)	540 (29.4)	51 (9.4)	1.00 (Ref)	0.597	
TCN2 766 CG	919 (50.0)	96 (10.8)	1.16 (1.16–0.26)	0.435	
TCN2 766 GG	380 (20.7)	41 (10.8)	1.12 (0.75–1.790)		
**Recessive Model**					0.682
TCN2 766 CC + CG (Ref)	1459 (79.3)	147 (10.1)	1.00 (Ref)		
TCN2 766 GG	380 (20.7)	41 (10.8)	1.08 (0.75–1.56)	0.682	

SGA, small-for-gestational-age. OR, odds ratio. Ref, reference category.

**Table 3:** Effect of Maternal Genotype—FAS Interaction on SGA Risk in New Zealand SCOPE Participants.

SNP	All *n* (%)	FAS Yes *n* (%) ^$^	FAS No *n* (%) ^$^	FAS Yes aOR (95% CI) *	FAS No aOR (95% CI) *	*p*-Value *
**MTHFR C677T**	1850 (100)					
CC (Ref)	887 (48.0)	628 (33.9)	259 (14.0)	1.00 (Ref)	1.87 (1.21–2.88)	0.072
CT	786 (42.5)	587 (31.7)	199 (10.8)	1.06 (0.71–1.56)	1.02 (0.58–1.77)	
TT	177 (9.6)	135 (7.3)	42 (2.3)	0.91 (0.46–1.78)	1.08 (0.37–3.12)	
CC (Ref)	887 (48.0)	628 (33.9)	259 (14.0)	1.00 (Ref)	1.87 (1.21–2.88)	0.019
CT + TT	963 (52.1)	722 (39.0)	241 (13.0)	1.03 (0.71–1.49)	1.03 (0.61–1.72)	
**MTHFR A1298C**	1869 (100)					
AA (Ref)	932 (49.9)	702 (37.6)	230 (12.3)	1.00 (Ref)	1.07 (0.65–1.75)	0.020
AC	778 (41.6)	560 (30.0)	218 (11.7)	0.86 (0.58–1.27)	1.13 (0.69–1.86)	
CC	159 (8.5)	101 (5.4)	58 (3.1)	0.90 (0.43–1.86)	2.92 (1.52–5.60)	
AA + AC (Ref)	1710 (91.5)	1262 (67.5)	448 (24.0)	1.00 (Ref)	1.17 (0.82–1.67)	0.005
CC	159 (8.5)	101 (5.4)	58 (3.1)	0.96 (0.47–1.95	3.11 (1.66–5.85)	
**MTHFD1 G1958A**	1870 (100)					
GG (Ref)	621 (33.2)	439 (23.5)	182 (9.7)	1.00 (Ref)	1.40 (0.82–2.38)	0.133
GA	920 (49.2)	680 (36.4)	240 (12.8)	1.01 (0.67–1.51)	1.02 (0.60–1.73)	
AA	329 (17.6)	245 (13.1)	84 (4.5)	0.69 (0.38–1.23)	1.84 (0.96–3.54)	
GG + GA (Ref)	1541 (82.4)	1119 (59.8)	422 (22.6)	1.00 (Ref)	1.18 (0.82–1.69)	0.062
AA	329 (17.6)	245 (13.1)	84 (4.5)	0.68 (0.40–1.16)	1.83 (1.00–3.36)	
**MTR A2756G**	1845 (100)					
AA (Ref)	1206 (65.4)	879 (47.6)	327 (17.7)	1.00 (Ref)	1.54 (1.03–2.30)	0.253
AG	569 (30.8)	415 (22.5)	154 (8.3)	1.34 (0.91–1.97)	1.60 (0.95–2.71)	
GG	70 (3.8)	53 (2.9)	17 (0.9)	1.37 (0.57–3.31)	0.67 (0.09–5.12)	
**MTRR A66G**	1860 (100)					
AA (Ref)	450 (24.2)	333 (17.9)	117 (6.2)	1.00 (Ref)	1.71 (0.92–3.18)	0.245
AG	892 (48.0)	650 (34.9)	242 (13.0)	1.01 (0.65–1.58)	1.08 (0.62–1.88)	
GG	518 (27.9)	375 (20.2)	143 (7.7)	0.85 (0.51–1.42)	1.44 (0.79–2.64)	
**TCN2 C776G**	1839 (100)					
CC (Ref)	540 (29.4)	401 (21.8)	139 (7.6)	1.00 (Ref)	1.23 (0.65–2.32)	0.057
CG	919 (50.0)	660 (35.9)	259 (14.1)	1.18 (0.77–1.81)	1.18 (0.70–2.00)	
GG	380 (20.7)	281 (15.3)	99 (5.4)	0.86(0.50 –1.50)	2.41 (1.31–4.41)	
CC + CG (Ref)	1459 (79.3)	1061 (57.7)	398 (21.6)	1.00 (Ref)	1.07 (0.74–1.57)	0.017
GG	380 (20.7)	281 (15.3)	99 (5.4)	0.77 (0.48–1.25)	2.16 (1.26–3.71)	

Ref, reference category. FAS, folic acid supplement use at 15 weeks’ gestation. * *p*-value for interaction effect. ^$^ Percentage of total number of participants genotyped for each SNP.

## Supplementary Materials

The following are available online at https://www.mdpi.com/2072-6643/12/6/1677/s1, Table S1: Details of Included Polymorphisms, Table S2: Maternal Genotype and Allele Frequency by Ethnicity in New Zealand SCOPE Participants.
