# Supplementary material for: The Effect of Interactions between Folic Acid Supplementation and One Carbon Metabolism Gene Variants on Small-for-Gestational-Age Births in the Screening for Pregnancy Endpoints (SCOPE) Cohort Study
Source: Nutrients. 2020 Jun 4;12(6):1677. doi: 10.3390/nu12061677 (PMC7352423; doi:10.3390/nu12061677)
Supplement: Supplementary file 1 [file nutrients-12-01677-s001.zip › Supplementary Table S1 Details of Included SNPs.docx]

**Supplementary Table 1. Details of Included Polymorphisms (data from [6, 33-40])**

| **Polymorphism**  **GenBank no.** | **Enzyme** | **Enzyme Function** | **Location** | **Amino Acid Substitution** | **Effect of Polymorphism** |
| --- | --- | --- | --- | --- | --- |
| **MTHFR 677**  **rs1801133**  AND  **MTHFR A1298C**  **rs1801131**  NM_005957 | 5, 10 Methylene-tetrahydrofolate reductase | Reduces 5, 10-MTHF to 5-MTHF, which donates a methyl group for the conversion of HC to methionine | 1p 36.3 | Ala to Val | MTHFR 677:   - Thermolabile enzyme - 677CT: 65% activity; 677TT: 30% activity Associated with lower red blood cell and plasma folate levels and increased homocysteine concentration - Lower DNA methylation status in TT homozygotes who have lower plasma folate levels   MTHFR 1298:   - Reduced folate levels - Associated with hypomethylation of DNA - Reduced enzyme activity |
| **MTR A2756G**  **rs1805087**  NM_000254 | Methionine Synthase | Catalyses the remethylation of homocysteine to methionine by transferring a methyl-group from 5-MTHF to homocysteine .  Converts folate to tetrahydrofolate | 1q43 | Asp to Gly | Polymorphism associated with elevated homocysteine |
| **MTRR A66G**  **rs1801394**  AF025794 | Methionine Synthase Reductase | Regenerates MTR to its functional form (active state) | 5p15.31 | Ile to Met | Wild-type homozygosity (AA) associated with higher homocysteine levels. |
| **MTHFD1 1958**  **rs2236225**  J04031 | Methylene tetrahydrofolate dehydrogenase | Catalyses the conversion of DHF through to 5,10-MTHF | 14q23.3 | Arg to Gln | Effects enzyme thermostability, diminishing DNA synthesis capacity |
| **TCN2**  **rs1801198**  NM_000355 | Transcobalamin II | Imports vitamin B12 which acts as an essential cofactor for MTR | Chr.22 | Arg to Pro | Lower levels of transcobalamin seen in homozygous GG variant (GG)  Influences homocysteine levels, with levels significantly higher in GG genotype vs wid type homozygous (CC) genotype |
