# Supplementary material for: The Effect of Interactions between Folic Acid Supplementation and One Carbon Metabolism Gene Variants on Small-for-Gestational-Age Births in the Screening for Pregnancy Endpoints (SCOPE) Cohort Study
Source: Nutrients. 2020 Jun 4;12(6):1677. doi: 10.3390/nu12061677 (PMC7352423; doi:10.3390/nu12061677)
Supplement: Supplementary file 1 [file nutrients-12-01677-s001.zip › Supplementary Table S2_Maternal Genotype and Allele Frequency by Ethnicity in New Zealand SCOPE Participants.docx]

**Supplementary Table 2: Maternal Genotype and Allele Frequency by Ethnicity in New Zealand SCOPE Participants**

| **SNP** | **Total**  **n (%)** | **NZ/Other European** | | **Māori** | | **Pacific** | | **Asian** | | **Indian** | | **Other Non- European** | | **p-value*** |
| --- | --- | --- | --- | --- | --- | --- | --- | --- | --- | --- | --- | --- | --- | --- |
| **MTHFR C677T** | 1850 (100) | n=1549 | | n=60 | | n=35 | | n=95 | | n=74 | | n=28 | | <0.001 |
| **Genotype:** |  |  |  |  |  |  |  |  |  |  |  |  |  |  |
| CC | 887 (48.0) | 718 | 46.1% | 30 | 50.0% | 26 | 74.3% | 49 | 51.6% | 54 | 73.0% | 10 | 35.7% |  |
| CT | 786 (42.5) | 678 | 43.5% | 27 | 45.0% | 8 | 22.9% | 39 | 41.1% | 19 | 25.7% | 15 | 53.6% |  |
| TT | 177 (9.6) | 162 | 10.4% | 3 | 5.0% | 1 | 2.9% | 7 | 7.4% | 1 | 1.4% | 3 | 10.7% |  |
| **Allele:** |  |  |  |  |  |  |  |  |  |  |  |  |  |  |
| C | 2560 (69.2) | 2114 | 67.8% | 87 | 72.5% | 60 | 85.7% | 137 | 72.1% | 127 | 85.8% | 35 | 62.5% |  |
| T | 1140 (30.8) | 1002 | 32.2% | 33 | 27.5% | 10 | 14.3% | 53 | 27.9% | 21 | 14.2% | 21 | 37.5% |  |
| **MTHFR A1298C** | 1869 (100) | n=1574 | | n=61 | | n=35 | | n=97 | | n=73 | | n=29 | | 0.068 |
| **Genotype:** |  |  |  |  |  |  |  |  |  |  |  |  |  |  |
| AA | 932 (49.9) | 768 | 48.8% | 36 | 59.0% | 22 | 62.9% | 60 | 61.9% | 31 | 42.5% | 15 | 51.7% |  |
| AC | 778 (41.6) | 664 | 42.2% | 21 | 34.4% | 11 | 31.4% | 33 | 34.0% | 36 | 49.3% | 13 | 44.8% |  |
| CC | 159 (8.5) | 142 | 9.0% | 4 | 6.6% | 2 | 5.7% | 4 | 4.1% | 6 | 8.2% | 1 | 3.4% |  |
| **Allele:** |  |  |  |  |  |  |  |  |  |  |  |  |  |  |
| A | 2642 (70.7) | 2200 | 69.9% | 93 | 76.2% | 55 | 78.6% | 153 | 78.9% | 98 | 67.1% | 43 | 74.1% |  |
| C | 1096 (29.3) | 948 | 30.1% | 29 | 23.8% | 15 | 21.4% | 41 | 21.1% | 48 | 32.9% | 15 | 25.9% |  |
| **MTHFD1 G1958A** | 1870 (100) | n=1575 | | n=61 | | n=35 | | n=97 | | n=73 | | n=29 | | <0.001 |
| **Genotype:** |  |  |  |  |  |  |  |  |  |  |  |  |  |  |
| GG | 621 (33.2) | 482 | 30.6% | 39 | 63.9% | 19 | 54.3% | 58 | 59.8% | 17 | 23.3% | 6 | 20.7% |  |
| GA | 920 (49.2) | 801 | 50.9% | 18 | 29.5% | 13 | 37.1% | 35 | 36.1% | 38 | 52.1% | 15 | 51.7% |  |
| AA | 329 (17.6) | 292 | 18.5% | 4 | 6.6% | 3 | 8.6% | 4 | 4.1% | 18 | 24.7% | 8 | 27.6% |  |
| **Allele:** |  |  |  |  |  |  |  |  |  |  |  |  |  |  |
| G | 2162 (57.8) | 1765 | 56.0% | 96 | 78.7% | 51 | 72.9% | 151 | 77.8% | 72 | 49.3% | 27 | 46.6% |  |
| A | 1578 (42.2) | 1385 | 44.0% | 26 | 21.3% | 19 | 27.1% | 43 | 22.2% | 74 | 50.7% | 31 | 53.4% |  |
| **SNP** | **Total**  **n (%)** | **NZ/Other European** | | **Māori** | | **Pacific** | | **Asian** | | **Indian** | | **Other Non- European** | | **p-value** |
| **MTR A2756G** | 1845 (100) | n=1553 | | n=61 | | n=34 | | n=95 | | n=74 | | n=28 | | <0.001 |
| **Genotype:** |  |  |  |  |  |  |  |  |  |  |  |  |  |  |
| AA | 1206 (65.4) | 1007 | 64.8% | 48 | 78.7% | 26 | 76.5% | 74 | 77.9% | 34 | 45.9% | 17 | 60.7% |  |
| GA | 569 (30.8) | 490 | 31.6% | 12 | 19.7% | 7 | 20.6% | 18 | 18.9% | 32 | 43.2% | 10 | 35.7% |  |
| GG | 70 (3.8) | 56 | 3.6% | 1 | 1.6% | 1 | 2.9% | 3 | 3.2% | 8 | 10.8% | 1 | 3.6% |  |
| **Allele:** |  |  |  |  |  |  |  |  |  |  |  |  |  |  |
| A | 2981 (80.8) | 2504 | 80.6% | 108 | 88.5% | 59 | 86.8% | 166 | 87.4% | 100 | 67.6% | 44 | 78.6% |  |
| G | 709 (19.2) | 602 | 19.4% | 14 | 11.5% | 9 | 13.2% | 24 | 12.6% | 48 | 32.4% | 12 | 21.4% |  |
| **MTRR A66G** | 1860 (100) | n=1564 | | n=61 | | n=35 | | n=97 | | n=74 | | n=29 | | <0.001 |
| **Genotype:** |  |  |  |  |  |  |  |  |  |  |  |  |  |  |
| AA | 450 (24.19) | 332 | 21.2% | 27 | 44.3% | 19 | 54.3% | 46 | 47.4% | 15 | 20.3% | 11 | 37.9% |  |
| AG | 892 (48.0) | 764 | 48.8% | 23 | 37.7% | 11 | 31.4% | 39 | 40.2% | 43 | 58.1% | 12 | 41.4% |  |
| GG | 518 (27.9) | 468 | 29.9% | 11 | 18.0% | 5 | 14.3% | 12 | 12.4% | 16 | 21.6% | 6 | 20.7% |  |
| **Allele:** |  |  |  |  |  |  |  |  |  |  |  |  |  |  |
| A | 1792 (48.2) | 1428 | 45.7% | 77 | 63.1% | 49 | 70.0% | 131 | 67.5% | 73 | 49.3% | 34 | 58.6% |  |
| G | 1928 (51.8) | 1700 | 54.3% | 45 | 36.9% | 21 | 30.0% | 63 | 32.5% | 75 | 50.7% | 24 | 41.4% |  |
| **TCN2 C766G** | 1839 (100) | n=1549 | | n=61 | | n=34 | | n=95 | | n=72 | | n=28 | | 0.457 |
| **Genotype:** |  |  |  |  |  |  |  |  |  |  |  |  |  |  |
| CC | 540 (29.4) | 470 | 30.3% | 20 | 32.8% | 8 | 23.5% | 18 | 18.9% | 16 | 22.2% | 8 | 28.6% |  |
| CG | 919 (50.0) | 764 | 49.3% | 33 | 54.1% | 21 | 61.8% | 50 | 52.6% | 25 | 34.7% | 16 | 57.1% |  |
| GG | 380 (20.7) | 315 | 20.3% | 8 | 13.1% | 5 | 14.7% | 27 | 28.4% | 31 | 43.1% | 4 | 14.3% |  |
| **Allele:** |  |  |  |  |  |  |  |  |  |  |  |  |  |  |
| C | 1999 (54.4) | 1704 | 55.0% | 73 | 59.8% | 37 | 54.4% | 86 | 45.3% | 57 | 39.6% | 32 | 57.1% |  |
| G | 1679 (45.6) | 1394 | 45.0% | 49 | 40.2% | 31 | 45.6% | 104 | 54.7% | 87 | 60.4% | 24 | 42.9% |  |

* p-value for overall genotype difference by ethnicity
